# The ectopic expression of Snail in MDBK cells does not induce epithelial-mesenchymal transition

**DOI:** 10.3892/ijmm.2015.2215

**Published:** 2015-05-19

**Authors:** GENYA IZAWA, WAKAKO KOBAYASHI, MISAKO HARAGUCHI, AKIHARU SUDO, MASAYUKI OZAWA

**Affiliations:** 1 Department of Biochemistry and Molecular Biology, Graduate School of Medical and Dental Sciences, Kagoshima University, Kagoshima 890-8544, Japan; 2 Department of Sport and Physical Education, Faculty of Physical Education, Kokushikan University, Tama-shi, Tokyo 206-8515, Japan

**Keywords:** epithelial-mesenchymal transition, Snail, Slug, zinc finger E-box-binding homeobox 1, Madin-Darby bovine kidney cells

## Abstract

Epithelial-mesenchymal transition (EMT), a key process in the tumor metastatic cascade, is characterized by the loss of cell-cell junctions and cell polarity, as well as by the acquisition of migratory and invasive properties. However, the precise molecular events that initiate this complex EMT process are poorly understood. Snail expression induces EMT in Madin-Darby canine kidney (MDCK) cells and the human epidermoid carcinoma cell line, A431. Snail is a zinc finger transcription factor and triggers EMT by suppressing E-cadherin expression. In the present study, to broaden our knowledge of Snail-induced EMT, we generated stable Snail transfectants using Madin-Darby bovine kidney (MDBK) cells. Contrary to the MDCK or A431 cells examined in our previous studies, the MDBK cells transfected with the Snail construct maintained an epithelial morphology and showed no sign of reduced cell-cell adhesiveness compared to the control cells. Consistent with these observations, the down-regulation of epithelial marker proteins, e.g. E-cadherin and desmoglein, and the upregulation of mesenchymal marker proteins, e.g., N-cadherin and fibronectin, were not detected. Furthermore, the E-cadherin promoter was not methylated. Therefore, in the MDBK cells, the ectopic expression of Snail failed to induce EMT. As previously demonstrated, in MDCK cells, Snail expression is accompanied by the increased expression of other EMT-inducing transcription factors, e.g., Slug and zinc finger E-box-binding homeobox 1 (ZEB1). However, the MDBK cells transfected with the Snail construct did not exhibit an increased expression of these factors. Thus, it is possible that the failure to upregulate other EMT-related transcription factors may explain the lack of Snail-mediated induction of EMT in MDBK cells.

## Introduction

Epithelial-mesenchymal transition (EMT) is a complex process through which epithelial cells lose their polarity and reorganize their cytoskeleton, while also acquiring a mesenchymal phenotype and increased motility (1,2). In addition to tissue remodeling, organ development and wound healing, EMT plays a critical role in cancer progression (3–6). The loss of a polarized epithelial phenotype and the acquisition of a mesenchymal phenotype endow cancer cells with the potential to invade and metastasize.

Epithelial cells are connected by the epithelial junctional complex, which consists of tight junctions, adherens junctions and desmosomes. E-cadherin is a component of the adherens junction and is involved in the formation and maintenance of epithelial structures (7). Desmoglein is a desmosome component and is expressed in desmosome-bearing epithelial cells (8). E-cadherin and desmoglein are members of the cadherin family of cell-cell adhesion molecules.

A hallmark of EMT is the loss of E-cadherin expression (9). Several transcription factors, including Snail, Slug, Twist and zinc finger E-box-binding homeobox 1 (ZEB1), have been implicated in the transcriptional repression of E-cadherin and the induction of EMT (9,10). Snail belongs to the Snail super-family of zinc finger transcription factors (11). Snail and Slug, a related superfamily member, are expressed during development in the early mesoderm and neural crest (12–14). These two zinc finger transcription factors repress E-cadherin transcription through an interaction of their C-terminal regions with a 5′-CACCTG-3′ sequence (termed an E-box) in the cadherin promoter (15,16). Correlative experiments have demonstrated that there is an inverse correlation between E-cadherin expression and Snail expression in human samples (17).

EMT is accompanied by epigenetic modifications, including DNA methylation (18,19). DNA methylation, which is commonly associated with gene repression and heterochromatin formation, is defined by the addition of a methyl group to the cytosine of a CpG dinucleotide in the promoter region of a gene (20). Transforming growth factor-β (TGF-β) is a multifunctional cytokine that regulates a broad range of cellular responses (21). TGF-β is the major mediator of EMT and induces the expression of Snail (22) and Slug (23). Recent studies have revealed that the effects of Snail on epithelial cells include the promotion of the expression of other EMT-inducing transcriptional factors, such as ZEB1 (24), and the activation of the TGF-β signaling pathway (25). Cells exposed to TGF-β undergo EMT, which includes E-cadherin promoter DNA methylation (26,27).

The ectopic expression of Snail in several epithelial cells, including Madin-Darby canine kidney (MDCK) cells and the human epidermoid carcinoma cell line, A431, has been shown to result in EMT (28,29). The precise molecular events that initiate the complex EMT process, however, are poorly understood. In the present study, in an aim to further understand the role of Snail in EMT, we generated stable Snail transfectants using a bovine cell line, Madin-Darby bovine kidney (MDBK) cells. Surprisingly, MDBK cells transfected with the Snail construct maintained their epithelial morphology and showed no sign of reduced cell-cell adhesiveness compared to the control cells. Consistent with these observations, the downregulation of the epithelial marker proteins, E-cadherin and desmoglein, and the upregulation of the mesenchymal marker proteins, N-cadherin and fibronectin, were not detected. Furthermore, the E-cadherin promoter was not methylated. Therefore, in the MDBK cells, the ectopic expression of Snail failed to induce EMT. Although Snail expression in MDCK cells is accompanied by the increased expression of other EMT-inducing transcription factors, such as Slug and ZEB1, MDBK cells ectopically expressing Snail did not show an increased expression of these factors. Thus, it seems that the inability to upregulate the expression of additional EMT-inducing transcription factors may explain the failure of ectopic Snail protein expression to induce EMT in MDBK cells.

## Materials and methods

### Cell lines and transfection

MDBK cells, a bovine kidney epithelial cell line and MDCK cells, a canine kidney epithelial cell line, provided by Dr Rolf Kemler (Max-Planck Institute of Immunobiology and Epigenetics, Freiburg, Germany) and Dr Satoshi Daikuhara (Kagoshima University), respectively. They were grown and were transfected as previously described (28) using the calcium phosphate method with 10 *µ*g of either plasmid DNA containing an HA-tagged human Snail construct (pC-SnailHA) or with a control empty vector containing a neomycin resistance gene.

### Antibodies

Mouse monoclonal antibodies (mAbs) against E-cadherin (Cat. no. 610182), p120 (Cat. no. 612537) and fibronectin (Cat. no. 610077) were purchased from BD Biosciences (Lexington, KY, USA). A mouse mAb against vimentin (Cat. no. 18-0052) was obtained from Zymed Laboratories (South San Francisco, CA, USA). Mouse mAbs recognizing Snail (Cat. no. 3895) and Slug (Cat. no. 9589) were purchased from Cell Signaling Technology (Danvers, MA, USA). A mAb against desmoglein 1 and 2 (Cat. no. 61002) was purchased from Progen Biotechnik GmbH (Heidelberg, Germany). A goat antibody recognizing ZEB1 (Cat. no. sc-5711) was purchased from Santa Cruz Biotechnology, Inc. (Santa Cruz, CA, USA). A mouse mAb recognizing vinculin (Cat. no. V9131) was purchased from Sigma-Aldrich (St. Louis, MO, USA). A rat mAb against hemagglutinin (HA; Cat. no. 11867423001) was purchased from Roche Applied Science (Mannheim, Germany). All secondary antibodies were obtained from Jackson ImmunoResearch Laboratories (West Grove, PA, USA).

### RT-PCR

Total RNA was extracted and reverse transcribed as previously described (29). The resulting cDNA was used as a template for PCR and the PCR conditions were optimized for each primer pair as previously described (29). The following primer combinations were used: E-cadherin sense, 5′-GACA CCCGATTCAAAGTGCAC-3′ and antisense, 5′-GTCTCTC TTCTGTCTCCTGAG-3′; Slug sense, 5′-GCGTTCTCCAGA CCCTGGT-3′ and antisense, 5′-GCACAGCAGCCAGACT CCT-3′; Twist1 sense, 5′-GAGTCCGCAGTCCTACGAG-3′ and antisense, 5′-TCTGTAGGACCTGGTAGAGG-3′; ZEB1 sense, 5′-TGGGCAGTGACGGTAGGTAT-3′ and antisense, 5′-GCA GGTCGAACCTCTTGATC-3′; and β-actin sense, 5′-CAA GGACCTCTACGCCAACA-3′ and antisense, 5′-CGTACTCC TGCTTGCTGATC-3′.

### Cell aggregation assay

Cell aggregation assays were performed as previously described (30). In brief, the cells were incubated for 10 min at 37°C in HEPES-buffered saline containing 0.01% trypsin (type XI; Sigma-Aldrich) and 2 mM CaCl_2_ or 1 mM EGTA. After the addition of soybean trypsin inhibitor (Sigma-Aldrich), the cells were washed, resuspended and incubated for 30 min at 37°C with constant rotation at 70 rpm. The extent of cell aggregation was represented by the index: (Nc-Np)/Nc, where Np and Nc are the total number of particles and cells/dish, respectively.

### Immunoblot analysis

For immunoblot analysis, proteins were separated by 8% polyacrylamide gel electrophoresis and transferred onto nitrocellulose membranes. After blocking, the membranes were incubated with specific primary antibodies followed by treatment with peroxidase-conjugated secondary antibodies (Jackson ImmunoResearch Laboratories). After washing with phosphate-buffered saline (PBS) containing 0.1% Tween-20, the protein bands were visualized by enhanced chemiluminescence (ECL; Amersham International, Little Chalfont, UK) as previously described (31). ImageJ software (National Institutes of Health) was used to quantify the protein levels. α-tubulin was used as a loading control.

### Immunofluorescence staining

For immunofluorescence, the cells were grown on coverslips, fixed with 3% paraformaldehyde in PBS for 20 min at room temperature, and permeabilized with 0.1% Triton X-100. The coverslips were immunostained with primary and secondary antibodies as previously described (31). To label the nuclei, 4′-6-diamidino-2-phenylindol (DAPI) was used. The cells were analyzed using an Olympus fluorescence microscope (Olympus, Tokyo, Japan) or a confocal laser scanning microscope (LSM 700; Carl Zeiss, Oberkochen, Germany).

### DNA methylation analysis

Genomic DNA (~0.75 *µ*g) was treated with sodium bisulfite using the EpiTect system (Qiagen, Germantown, MD, USA). The bisulfite-converted DNA (~400 ng) was used as a template for PCR amplification of the CpG islands in the CDH1 promoter. The primer pairs were sense, 5′-GAGA TTTGAAGTTTAAAAGATAGAA-3′ and antisense, 5′-AAC TAAAATCTAACAAAACTTCTAC-3′. PCR products were purified on a 1.5% agarose gel using a Gel Extraction kit (Qiagen) and cloned into the pGEM-T easy vector (Promega, Madison, WI, USA). Four or five randomly selected clones from each sample were selected for sequencing. As a positive control for methylated DNA, genomic DNA was methylated *in vitro* using CpG methyltransferase (M.SssI; New England BioLabs, Inc., Ipswich, MA, USA).

## Results

### The ectopic expression of Snail does not induce morphological changes or change the adhesiveness of MDBK cells

**Figure 1:**
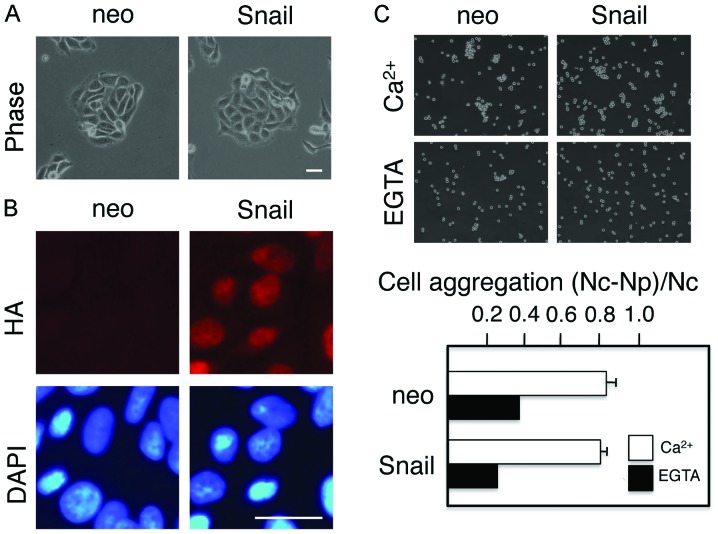
Madin-Darby bovine kidney (MDBK) cells ectopically expressing Snail protein display characteristics of the epithelial phenotype. (A) Both the control MDBK cells transfected with an empty vector containing a neomycin resistance gene (neo) and MDBK cells transfected with an expression vector encoding HA-tagged Snail protein (Snail) displayed typical epithelial cell morphology. (B) Immunofluorescence staining with an anti-HA antibody revealed the protein expression of Snail in the nucleus, which was co-stained with DAPI. (C) Cell aggregation assays revealed that the cells ectopically expressing Snail protein had similar adhesive properties as the control (neo) cells; furthermore, the observed cell-cell adhesion was calcium-dependent, indicating that it was mediated by cadherins. Scale bars, 20 *µ*m.

MDBK cells, a cell line derived from bovine kidney, display epithelial properties, including a brickstone morphology. We introduced a control empty vector containing a neomycin resistance gene or an expression vector encoding HA-tagged Snail protein into the MDBK cells and isolated stable transfectants, designated as neo or Snail cells, respectively. The Snail cells retained the same epithelial morphology as the control neo cells (Fig. 1), despite the clear nuclear localization of Snail protein, as revealed by staining with an anti-HA antibody (Fig. 1B). Thus, contrary to our previous experiments with MDCK or A431 cells (28,29), the ectopic expression of Snail did not induce morphological changes that were characteristic of EMT.

Cells undergoing EMT lose cell-cell adhesion. It is well known that cadherins at the cell surface resist tryptic digestion in the presence of Ca^2+^, but not in the absence of Ca^2+^ (7). Therefore, cell aggregation assays following the tryptic digestion of cells in the presence of either 2 mM Ca^2+^ or 1 mM EGTA can be used to distinguish between cadherin-mediated and cadherin-independent cell-cell adhesion. Cell aggregation assays revealed Ca^2+^-dependent, cadherin-mediated cell-cell adhesion in both the neo cells and Snail cells; no significant differences in cell-cell adhesion were observed between these two cell populations (Fig. 1C) These results are consistent with the morphological observation that the Snail cells were not undergoing EMT.

### The ectopic expression of Snail in MDBK cells does not alter the expression levels of epithelial and mesenchymal markers

**Figure 2:**
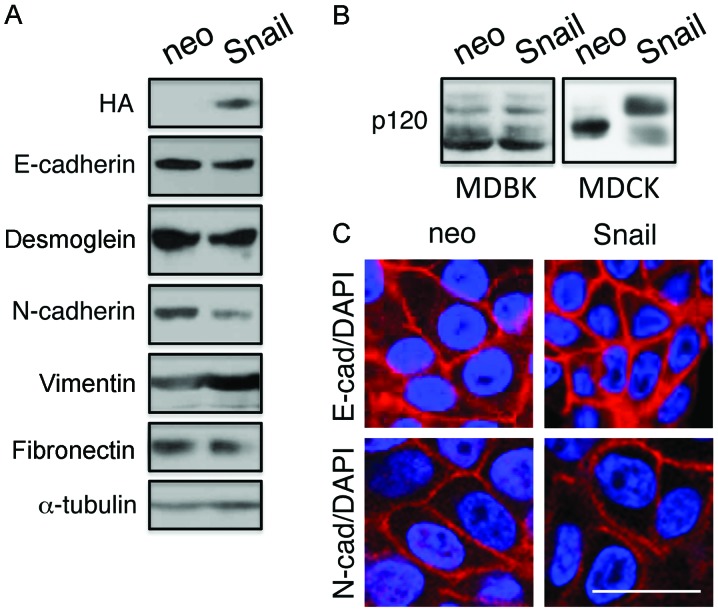
Epithelial-mesenchymal transition (EMT) is not induced in Madin-Darby bovine kidney (MDBK) cells ectopically expressing Snail. (A) Immunoblot analysis revealed that Snail expression in the MDBK cells did not decrease the expression of the epithelial markers, E-cadherin and desmoglein, and did not increase the expression of the mesenchymal markers, N-cadherin, vimentin and fibronectin. α-tubulin was used as a loading control. (B) The ectopic expression of Snail altered the splicing patterns of p120 in the Madin-Darby canine kidney (MDCK) cells, but not in the MDBK cells. (C) E-cadherin (E-cad) and N-cadherin (N-cad) were detected at the membrane of the control cells [transfected with a neomycin resistance gene (neo) cells] and in the cells ectopically expressing Snail protein (Snail). Cells were stained with the appropriate primary antibody followed by a rhodamine-labeled secondary antibody. DAPI was used to detect the nucleus. Scale bar, 20 *µ*m.

Next, we determined the expression levels of epithelial markers, E-cadherin and desmoglein, using immunoblot analysis (Fig. 2). Although the Snail cells expressed exogenous Snail protein as detected by anti-HA antibodies, they showed essentially the same expression levels of E-cadherin and desmoglein as the control neo cells. The control neo cells also expressed the mesenchymal markers, N-cadherin, vimentin and fibronectin, and the expression levels of these proteins did not increase in the Snail cells (Fig. 2 and Table I). Thus, the ectopic expression of Snail in the MDBK cells did not lead to the downregulation of E-cadherin or desmoglein expression or to the upregulation of N-cadherin, vimentin or fibronectin expression. Furthermore, as previously reported (28), the expression of Snail altered the splicing patterns of p120 in the MDCK cells, but not in the MDBK cells (Fig. 2B).

Consistent with the observations that Snail expression did not alter cadherin-mediated cell-cell adhesion (Fig. 1) or the expression levels of E- or N-cadherin (Fig. 2), immunofluores-cence staining revealed that E- and N-cadherin were detected at the plasma membrane of both the neo and Snail cells (Fig. 2C).

### The E-cadherin promoter is not methylated in MDBK cells ectopically expressing Snail protein

**Figure 3:**
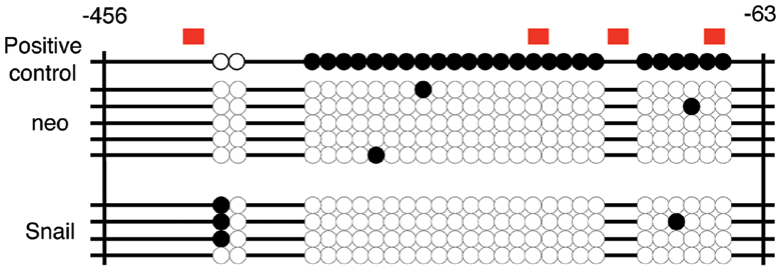
Ectopic expression of Snail in Madin-Darby bovine kidney (MDBK) cells does not induce DNA methylation of the E-cadherin promoter. Diagram showing the position of 4 E-boxes (-403 to -398, -201 to -196, -151 to -146, and -100 to -95; red bars) and CpG dinucleotides within the E-cadherin promoter region (circles). Genomic DNA was isolated from the control cells [transfected with a neomycin resistance gene (neo) cells] and Snail cells (cells ectopically expressing Snail protein), and the methylation of the E-cadherin promoter was analyzed by bisulfite sequencing. Genomic DNA incubated with CpG methyl-transfease prior to bisulfite treatment was used as a positive control for methylated DNA. Methylated and unmethylated dinucleotides are indicated as filled and open circles, respectively.

Previous analysis of the E-cadherin gene revealed that its proximal promoter contains CpG islands, which are targets for methylation during TGF-β-induced EMT (26,27). Therefore, in this study, we examined the methylation status of the E-cadherin promoter. No significant *de novo* DNA methylation was detected at the E-cadherin promoter in the Snail cells as compared to the control neo cells, as measured by bisulfite sequencing (Fig. 3). These results were consistent with the observation that no significant downregulation of E-cadherin expression was detected in the Snail cells.

### The ectopic expression of Snail protein in MDBK cells does not increase the production of EMT-related transcription factors

**Figure 4:**
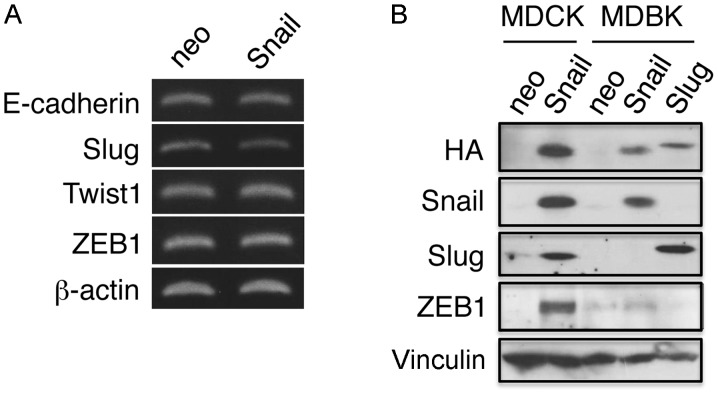
(A) RT-PCR of Slug, Twist and ZEB1 mRNA in control cells [transfected with a neomycin resistance gene (neo) cells] and Snail cells (cells ectopically expressing Snail protein). β-actin was used as an internal control. No significant differences were observed between the control cells and the Snail cells with respect to the mRNA levels of these proteins. (B) Immunoblot analysis using anti-Slug and anti-ZEB1 antibodies. Vinculin served as a loading control. Ectopic Snail expression increased Slug and ZEB1 protein levels in Madin-Darby canine kidney (MDCK) cells, but not in Madin-Darby bovine kidney (MDBK) cells. Ectopic expression of Snail in MDBK cells slightly increased the expression level of ZEB1 protein, but the quantification of the blots using NIH ImageJ software revealed that the relative amounts of ZEB1 protein in Snail-MDBK cells were <20% of those in the Snail-MDCK cells.

As previously reported, the expression of lymphoid enhancer-binding factor 1 (LEF-1), an EMT-inducer, in MDCK cells resulted in the significantly increased expression of other EMT-inducing transcription factors, including Slug and ZEB1 (31). Using an Agilent Whole Canine Genome microarray, we found that the ectopic expression of Snail in MDCK cells resulted in the increased expression of Slug and ZEB1 [Ozawa *et al*, (32)]. The upregulation of Twist and ZEB1 expression and the induction of EMT in human mammary epithelial HMLE cells upon Snail overexpression have been previously reported (33). Therefore, in this study, we used RT-PCR to compare the mRNA expression levels of Slug, Twist and ZEB1 in the neo cells and Snail cells. We observed no significant changes in the mRNA levels of these factors upon the ectopic expression of Snail (Fig. 4). Furthermore, immunoblot analysis revealed that MDCK cells expressing Snail presented with an increased Slug and ZEB1 production at the protein level, whereas the MDBK cells expressing Snail did not. Thus, our data suggest that the Snail-mediated upregulation of Slug and ZEB1 is required for the downregulation of E-cadherin expression and the induction of EMT.

## Discussion

In this study, we demonstrated that the ectopic expression of Snail in MDBK cells, a bovine kidney epithelial cell line, failed to induce changes that were characteristic of EMT. None of the following events were observed: i) epithelial to fibroblastic morphological changes; ii) reduced cell-cell adhesion; iii) the downregulation of the epithelial markers, E-cadherin and desmoglein; or iv) the upregulation of the mesenchymal markers, N-cadherin, vimentin and fibronectin. Although the downregulation of E-cadherin and desmoglein in human squamous cell carcinoma HSC-4 cells is not extensive (34), the transfection of cells with the Snail construct used in the present study has been shown to induce EMT in a number of cell lines of different origin, including canine kidney epithelial MDCK cells (28,29), the human epidermoid carcinoma cell line, A431 (8,29), the human squamous cell carcinoma cell line, HSC5 (35) and the murine embryonal carcinoma cell, P19 (Izawa *et al*, unpublished data).

The exogenous expression of Snail has been reported to suppress the activity of an E-cadherin promoter-reporter construct in MDCK cells, but not in mouse mammary epithelial NMuMG cells (36). In that study, the reason behind the cell context-dependent Snail activity was not analyzed. Snail protein undergoes post-translational modifications, including glycogen synthase kinase-3 (GSK3β)-mediated phosphorylation (37), and protein kinase D1 (PKD1)-mediated phosphorylation (38), followed by ubiquitination, which leads to Snail protein degradation. In a previous study, although wild-type Snail protein could not induce EMT in MCF7 cells, mutant Snail protein, in which serine residues that are targets for GSK3β phosphorylation were substituted with alanine residues, was stabilized and did induce EMT (37). Therefore, the failure of Snail protein to induce EMT in MCF7 cells was explained by its rapid turnover rate and low protein expression in this cell line (37). Since the protein levels of Snail in MDBK cells were very similar/comparable (>70%) to those in MDCK cells (Fig. 4B), it seems less likely that rapid turnover and low protein levels were responsible for the failure of Snail protein to induce EMT in MDBK cells. Consistent with this hypothesis, the addition of the GSK3β inhibitor, 6-bromoindirubin-3′-oxime (BIO), did not induce EMT in MDBK cells ectopically expressing Snail (Izawa *et al*, unpublished data). Phosphorylation regulates the subcellular localization of Snail protein (39). In this study, the immunostaining of Snail, however, revealed that a significant portion of Snail is present in the nucleus (Fig. 1B).

The levels of EMT-inducing transcription factors are under the control of microRNAs, which are regulated by wild-type p53 (40,41). Therefore, the presence of wild-type p53 has been proposed to be responsible for the failure of overexpressed Snail protein to induce EMT in MCF7 cells (33). MDBK cells seem to express wild-type p53 (42). Thus, the same mechanism could be operating in MDBK cells to suppress Snail activity. However, MDCK cells, in which the overexpression of Snail does induce EMT, also express wild-type p53 (43). Therefore, the presence of wild-type p53 alone cannot explain the failure of Snail to induce EMT in some cell lines.

As previously reported, the expression of LEF-1, an EMT-inducer, in MDCK cells resulted in the significantly increased expression of other EMT-inducing transcription factors, e.g., Slug and ZEB1 (31). The upregulation of Twist and ZEB1 expression and the induction of EMT in HMLE cells upon Snail overexpression have also been reported (33). Therefore, the expression of multiple EMT-inducing factors seems to be necessary to complete the EMT process. As demonstrated in the present study, ectopic Snail expression increased Slug and ZEB1 production at the protein level in MDCK cells, but not in the MDBK cells. Double transfectants of MDBK cells expressing Snail and Slug showed no sign of EMT (Izawa *et al*, unpublished data). Thus, the failure to upregulate multiple EMT-inducing factors may underlie the inability of the ectopic expression of Snail to induce EMT in MDBK cells.

It has been demonstrated that shRNA-mediated knockdown of E-cadherin induces EMT (44). Thus, the knockdown of E-cadherin expression seems to be an essential step for the induction of EMT. Although the suppression of E-cadherin expression during EMT is commonly associated with CpG island methylation within its promoter, our bisulfite sequencing analysis revealed that the E-cadherin promoter was not methylated in MDBK cells ectopically expressing Snail protein, and immunoblot analysis revealed that E-cadherin expression was maintained in those cells. Therefore, the failure to downregulate E-cadherin expression may also explain why Snail-expressing MDBK cells did not undergo EMT.

## Figures and Tables

**Table I:** Relative expression levels of epithelial and mesenchymal markers in MDBK cells ectopically expressing Snail protein.

	E-cadherin	Desmoglein	N-cadherin	Fibronectin	Vimentin
Ratios	0.76±0.09	0.87±0.06	0.74±0.13	0.74±0.13	1.16±0.12

The expression levels were determined using ImageJ software (National Institutes of Health). The data are presented as the relative intensity of the bands in the Snail-transfected MDBK cell samples as compared to the control [transfected with a neomycin resistance gene (neo)] MDBK cell samples. Values are the means ± SE obtained from 3 independent clones. MDBK cells, Madin-Darby bovine kidney cells.

## Abbreviations

EMT: epithelial-mesenchymal transition
MDBK: Madin-Darby bovine kidney
MDCK: Madin-Darby canine kidney

## References

[1] Duband JL, Monier F, Delannet M, Newgreen D (1995). Epithelium-mesenchyme transition during neural crest development. Acta Anat (Basel).

[2] Hay ED (1995). An overview of epithelio-mesenchymal transformation. Acta Anat (Basel).

[3] Thiery JP (2002). Epithelial-mesenchymal transitions in tumour progression. Nat Rev Cancer.

[4] Huber MA, Kraut N, Beug H (2005). Molecular requirements for epithelial-mesenchymal transition during tumor progression. Curr Opin Cell Biol.

[5] Christiansen JJ, Rajasekaran AK (2006). Reassessing epithelial to mesenchymal transition as a prerequisite for carcinoma invasion and metastasis. Cancer Res.

[6] Nawshad A, Lagamba D, Polad A, Hay ED (2005). Transforming growth factor-beta signaling during epithelial-mesenchymal transformation: Implications for embryogenesis and tumor metastasis. Cells Tissues Organs.

[7] Takeichi M (1988). The cadherins: Cell-cell adhesion molecules controlling animal morphogenesis. Development.

[8] Koch PJ, Walsh MJ, Schmelz M, Goldschmidt MD, Zimbelmann R, Franke WW (1990). Identification of desmoglein, a constitutive desmosomal glycoprotein, as a member of the cadherin family of cell adhesion molecules. Eur J Cell Biol.

[9] Thiery JP, Acloque H, Huang RY, Nieto MA (2009). Epithelial-mesenchymal transitions in development and disease. Cell.

[10] Kalluri R, Weinberg RA (2009). The basics of epithelial-mesenchymal transition. J Clin Invest.

[11] Nieto MA (2002). The snail superfamily of zinc-finger transcription factors. Nat Rev Mol Cell Biol.

[12] Barrallo-Gimeno A, Nieto MA (2005). The Snail genes as inducers of cell movement and survival: Implications in development and cancer. Development.

[13] Peinado H, Olmeda D, Cano A (2007). Snail, Zeb and bHLH factors in tumour progression: An alliance against the epithelial phenotype?. Nat Rev Cancer.

[14] Cobaleda C, Pérez-Caro M, Vicente-Dueñas C, Sánchez-García I (2007). Function of the zinc-finger transcription factor SNAI2 in cancer and development. Annu Rev Genet.

[15] Cano A, Pérez-Moreno MA, Rodrigo I, Locascio A, Blanco MJ, del Barrio MG, Portillo F, Nieto MA (2000). The transcription factor snail controls epithelial-mesenchymal transitions by repressing E-cadherin expression. Nat Cell Biol.

[16] Batlle E, Sancho E, Francí C, Domínguez D, Monfar M, Baulida J, García De Herreros A (2000). The transcription factor snail is a repressor of E-cadherin gene expression in epithelial tumour cells. Nat Cell Biol.

[17] Côme C, Magnino F, Bibeau F, De Santa Barbara P, Becker KF, Theillet C, Savagner P (2006). Snail and slug play distinct roles during breast carcinoma progression. Clin Cancer Res.

[18] Cedar H, Bergman Y (2009). Linking DNA methylation and histone modification: Patterns and paradigms. Nat Rev Genet.

[19] Reik W (2007). Stability and flexibility of epigenetic gene regulation in mammalian development. Nature.

[20] McCabe MT, Brandes JC, Vertino PM (2009). Cancer DNA methylation: Molecular mechanisms and clinical implications. Clin Cancer Res.

[21] Derynck R, Zhang YE (2003). Smad-dependent and Smad-independent pathways in TGF-β family signalling. Nature.

[22] Peinado H, Quintanilla M, Cano A (2003). Transforming growth factor β-1 induces snail transcription factor in epithelial cell lines: Mechanisms for epithelial mesenchymal transitions. J Biol Chem.

[23] Romano LA, Runyan RB (2000). Slug is an essential target of TGFbeta2 signaling in the developing chicken heart. Dev Biol.

[24] Dave N, Guaita-Esteruelas S, Gutarra S, Frias À, Beltran M, Peiró S, de Herreros AG (2011). Functional cooperation between Snail1 and twist in the regulation of ZEB1 expression during epithelial to mesenchymal transition. J Biol Chem.

[25] Dhasarathy A, Phadke D, Mav D, Shah RR, Wade PA (2011). The transcription factors Snail and Slug activate the transforming growth factor-beta signaling pathway in breast cancer. PLoS One.

[26] Yang X, Pursell B, Lu S, Chang TK, Mercurio AM (2009). Regulation of β4-integrin expression by epigenetic modifications in the mammary gland and during the epithelial-to-mesenchymal transition. J Cell Sci.

[27] Dong C, Wu Y, Yao J, Wang Y, Yu Y, Rychahou PG, Evers BM, Zhou BP (2012). G9a interacts with Snail and is critical for Snail-mediated E-cadherin repression in human breast cancer. J Clin Invest.

[28] Ohkubo T, Ozawa M (2004). The transcription factor Snail downregulates the tight junction components independently of E-cadherin downregulation. J Cell Sci.

[29] Haraguchi M, Okubo T, Miyashita Y, Miyamoto Y, Hayashi M, Crotti TN, McHugh KP, Ozawa M (2008). Snail regulates cell-matrix adhesion by regulation of the expression of integrins and basement membrane proteins. J Biol Chem.

[30] Ozawa M, Ringwald M, Kemler R (1990). Uvomorulincatenin complex formation is regulated by a specific domain in the cytoplasmic region of the cell adhesion molecule. Proc Natl Acad Sci USA.

[31] Kobayashi W, Ozawa M (2013). The transcription factor LEF-1 induces an epithelial-mesenchymal transition in MDCK cells independent of β-catenin. Biochem Biophys Res Commun.

[32] Ozawa M, Kobayashi W (2015). Reversibility of the Snail-induced epithelial-mesenchymal transition revealed by the CreloxP system. Biochem Biophys Res Commun.

[33] Zhang P, Wei Y, Wang L, Debeb BG, Yuan Y, Zhang J, Yuan J, Wang M, Chen D, Sun Y (2014). ATM-mediated stabilization of ZEB1 promotes DNA damage response and radioresistance through CHK1. Nat Cell Biol.

[34] Kume K, Haraguchi M, Hijioka H, Ishida T, Miyawaki A, Nakamura N, Ozawa M (2013). The transcription factor Snail enhanced the degradation of E-cadherin and desmoglein 2 in oral squamous cell carcinoma cells. Biochem Biophys Res Commun.

[35] Shimokawa M, Haraguchi M, Kobayashi W, Higashi Y, Matsushita S, Kawai K, Kanekura T, Ozawa M (2013). The transcription factor Snail expressed in cutaneous squamous cell carcinoma induces epithelial-mesenchymal transition and down-regulates COX-2. Biochem Biophys Res Commun.

[36] Shirakihara T, Saitoh M, Miyazono K (2007). Differential regulation of epithelial and mesenchymal markers by deltaEF1 proteins in epithelial mesenchymal transition induced by TGF-β. Mol Biol Cell.

[37] Zhou BP, Deng J, Xia W, Xu J, Li YM, Gunduz M, Hung MC (2004). Dual regulation of Snail by GSK-3β-mediated phosphorylation in control of epithelial-mesenchymal transition. Nat Cell Biol.

[38] Zheng H, Shen M, Zha YL, Li W, Wei Y, Blanco MA, Ren G, Zhou T, Storz P, Wang HY (2014). PKD1 phosphorylation-dependent degradation of SNAIL by SCF-FBXO11 regulates epithelial-mesenchymal transition and metastasis. Cancer Cell.

[39] Domínguez D, Montserrat-Sentís B, Virgós-Soler A, Guaita S, Grueso J, Porta M, Puig I, Baulida J, Francí C, García de Herreros A (2003). Phosphorylation regulates the subcellular location and activity of the snail transcriptional repressor. Mol Cell Biol.

[40] Chang CJ, Chao CH, Xia W, Yang JY, Xiong Y, Li CW, Yu WH, Rehman SK, Hsu JL, Lee HH (2011). p53 regulates epithelial-mesenchymal transition and stem cell properties through modulating miRNAs. Nat Cell Biol.

[41] Kim T, Veronese A, Pichiorri F, Lee TJ, Jeon YJ, Volinia S, Pineau P, Marchio A, Palatini J, Suh SS (2011). p53 regulates epithelial-mesenchymal transition through microRNAs targeting ZEB1 and ZEB2. J Exp Med.

[42] Devireddy LR, Jones CJ (1999). Activation of caspases and p53 by bovine herpesvirus 1 infection results in programmed cell death and efficient virus release. J Virol.

[43] Zhang Y, Yan W, Chen X (2013). Mutant p53 cooperates with knockdown of endogenous wild-type p53 to disrupt tubulogenesis in Madin-Darby canine kidney cells. PLoS One.

[44] Onder TT, Gupta PB, Mani SA, Yang J, Lander ES, Weinberg RA (2008). Loss of E-cadherin promotes metastasis via multiple downstream transcriptional pathways. Cancer Res.

